# Dataset of response characteristics of H_2_-producing bacteria consortium to β-lactams, aminoglycosides, macrolides, quinolones antibiotics

**DOI:** 10.1016/j.dib.2022.108354

**Published:** 2022-06-06

**Authors:** Dong Xiao, Hailun He, Xiaoxin Yan, Mohamed Keita, Norberto Daniel Diaz, Dayong Chen, Jing Ma, Yidong Zhang, Jin Li, Essono Oyono Julien, Xiaotao Yan

**Affiliations:** a CUMT-UCASAL Joint Research Center for Biomining and Soil Ecological Restoration, State Key Laboratory of Coal Resources and Safe Mining, China University of Mining and Technology, Xuzhou, Jiangsu province 221116 China; b School of life science, Central South University, Changsha, Hunan 410083, China; c Xiangya School of Medicine, Central South University, Changsha, Hunan 410083, China; d CUMT-UCASAL Joint Research Center for Biomining and Soil Ecological Restoration, Universidad Católica de Salta, Salta A4400EDD, Argentina; e School of Environment Science and Spatial Informatics, China University of Mining and Technology, Xuzhou, Jiangsu province 221116 China; f Xuzhou No.1 Peoples Hospital, Xuzhou, Jiangsu province 221116, China

**Keywords:** Renewable energy, Bio-hydrogen, β-lactams antibiotic, Penicillin G, Cefaclor, Anaerobic fermentation, Coal geological microbiology

## Abstract

Antibiotics on H_2_ producing bacteria shall be considered as being one of the critical elements in biological H_2_ production utilizing livestock manure as raw resources. Despite the fact that the manure stands a significance role in bio-fermentation, the possibility of antibiotics being contained in excreta shall not be eliminated. Findings of whether the above saying might threaten the safety of bio-H_2_ production needs to be further studied. The experiment subjects include: six single and three combined antibiotics were tested and analyzed by the application of the gradient experiment method. Along with the H_2_ production rate, CHO content, pH and OD_600_ were used to analyze the effects of various antibiotics introduction on the hydrolysis, fermentation and H_2_ production. To a further extent, four typical representative samples were selected for biodiversity analysis from the single antibiotic experiment groups. Amounting more than 6000 pieces of data were obtained in a series of experiments. Data suggested that remarkable measure of antibiotics have various degrees of H_2_ production inhibition, while some antibiotics, Penicillin G, Streptomycin Sulfate, and their compound antibiotics, could promote the growth of *Ethanoligenens* sp. and improve H_2_ yield in the contrary. Correspondent to the transition of key metabolic intermediates and end products, the mechanism of each antibiotic type and dose on H_2_ production were summarized as follows: the main inhibitory mechanisms were: (1) board-spectrum inhibition, (2) partial inhibition, (3) H_2_ consumption enhancement; and the enhancement mechanisms were: (1) enhance the growth of H_2_-producing bacteria, (2) enhanced starch hydrolysis, (3) inhibitory H_2_ consumption or release of acid inhibition. Meanwhile, data analysis found that the effect of antibiotics on H_2_ producing was not only related to type, but also to dosage. Even one kind of antibiotic may have completely opposite effects on H_2_-producing bacteria under different dosage conditions. Inhibition of H_2_ yield was highest with Levofloxacin at 6.15 mg/L, gas production was reduced by 88.77%; and enhancement of H_2_ yield was highest with Penicillin G at 7.20 mg/L, the gas production increased by 72.90%.

**Specifications Table**

**Table:** 

Subject	Renewable Energy, Sustainability and the Environment
Specific subject area	Response of anthracite H_2_-producing bacteria consortium to Penicillin G and Cefaclor
Type of data	Table and Figure
How the data were acquired	H_2_ yield data via gas needle and gas chromatography (7890A, Agilent, America); CHO and OD_600_ data via spectrophotometer (BioMate 3S, Thermo Scientific, America); pH value via electronic pH meter (Star A211, Orion, America). H_2_-producing bacteria community structure data via high-pass sequencing Raw fastq files were demultiplexed, quality-filtered by Trimmomatic and Merged by FLASH Operational taxonomic units (OTUs) were clustered with 97% similarity cutoff using UPARSE (version7.1 http://drive5.com/uparse/) and chimeric sequences were identified and removed using UCHIME. The taxonomy of each 16S rRNA gene sequence was analyzed by RDP Classifier algorithm (http://rdp.cme.msu.edu/) against the Silva (SSU123) 16S rRNA database using confidence threshold of 70%. Bacterial Galanz-stained photographs were taken through BX43 microscope (BX43, Olympus, Japan).
	Community bar chart: R language (version 3.3.1) tool. Evolutionary tree: Mega (version 10.0). Heatmap: R language (version 3.3.1) vegan package. Ternary analysis: GGTERN. RDA-CCA analysis: R language (version 3.3.1) rda or cca analysis and graphing in the vegan package. Spearman correlation heatmap: R (version 3.3.1) (pheatmap package). Circos chart: Circos-0.67-7.
Data format	Raw Analyzed
Description of data collection	The effects of different type and dosage of antibiotics on H_2_-producing bacteria were determined by gradient experiment.
Data source location	Anthracite H_2_-producing bacteria consortium were collected from Zhaozhuang Mining (GPS coordinates is 35°34′10″N,112°53′55″E).
Data accessibility	Repository name: Mendeley Data Data identification number: 10.17632/vgb4rcsspf.3 Direct URL to data: https://data.mendeley.com/datasets/vgb4rcsspf/draft?a=56945b77-bb3c-4871-aa6f-f3afb5fc972e The 16S rRNA gene sequences were deposited in the NCBI Sequence Read Archive under accession number PRJNA784035.
Related research article	D. Xiao, H. He, X. Yan, N.D. Diaz, D. Chen, J. Ma, Y. Zhang, J. Li, M. Keita, E.O. Julien, X. Yan, The response regularity of biohydrogen production by anthracite H_2_-producing bacteria consortium to six conventional veterinary antibiotics, J. Environ. Manage. 315 (2022) 115088. https://doi.org/10.1016/j.jenvman.2022.115088.

## Value of the Data

• Datasets proven various types of antibiotic effects and dosage, mainly reflecting on the key intermediate metabolites and production capacity of the H_2_-producing bacteria consortium.
• Clearly showing the types and doses of antibiotics that can promote and inhibit hydrogen production.
• It provides basic data for revealing the changes of microbial community diversity and the mechanism of metabolic cooperation among bacteria under the influence of antibiotics.
• It can be used in many different types of studies focusing on bio-hydrogen production from agricultural waste.
• The data can provide support for researchers to study the industrialization of anaerobic digestion and the metabolic pathway of microbial H_2_ fermentation process.

## 1 Data Description

Datasets could be referred as supplementary data, consist of 10 tables and 9 figures. Table 1 contains two types of key experimental design information. (1) Single antibiotics and combinations of combination antibiotics were used in the experiments. The former includes 6 kinds of 4 categories; the latter includes 3 groups of compound antibiotics. (2) Dosage design of gradient experiment for each antibiotic or compound antibiotics. Dosage gradient gradually decreased from 100.00 % to 1.56 % by dichotomy.

**Table 1:** Experiment design of the corresponding relationship between each antibiotic gradient and dosage

				**Amount Dosage Corresponding to GP (mg/L)**							
**Group**	**Category**	**Antibiotic**	**Abbreviation**	**100.00%**	**50.00%**	**25.00%**	**12.50%**	**6.25%**	**3.13%**	**1.56%**	**0.00%**
**Signal**	β-lactams	Penicillin G	P	55.38	27.69	13.85	6.92	3.46	1.73	0.86	0.00
		Cefaclor	C	15.38	7.69	3.85	1.92	0.96	0.48	0.24	0.00
	Aminogly-cosides	Streptomycin Sulfate	S	30.77	15.39	7.69	3.85	1.92	0.96	0.48	0.00
		Amikacin Sulfate	A	15.00	7.50	3.75	1.88	0.94	0.47	0.23	0.00
	Macrolides	Erythromycin	E	123.08	61.54	30.77	15.39	7.69	3.85	1.92	0.00
	Quinolones	Levofloxacin	L	6.15	3.08	1.54	0.77	0.38	0.19	0.10	0.00

**Compound**	Penicillin G + Streptomycin Sulfate		P-S	√	○	√	√	√	○	○	√
	Amikacin Sulfate + Streptomycin Sulfate		A-S	√	○	√	√	√	○	○	√
	Levofloxacin + Cefaclor		L-C	√	○	√	√	√	○	○	√

√: select; ○: not select.

Raw and analytical data of H_2_ yield changes due to the application of treatment on various dosage of single antibiotics and compound antibiotics were recorded in Tables 2 and 3 respectively. Each treatment consists of 5 parallel samples. With reference to the usb-table “Gas yield Raw data” of Tables 2 and 3, T_Gas could be defined as the total gas yield amount and the unit generally referred as mL/Sample. Furthermore, C_H_2_ is construed as the H_2_ concentration of each sample in terms of terminology written as %VOL;. V_H_2_ refer to the H_2_ yield amount and interpret as the mL/Sample. In terms of ST_H_2_, it is the average H_2_ production rate and the unit is mL/Treatment; Mm_H_2_ is the substrate molar H_2_ production rate the unit is mM/g. “ST” sub-table is the statistics of the substrate molar H_2_ production rate in the “Gas yield Raw data” sub-table. The above table is being prepared for the intention of graphing. The “ST-analysis” sub-table records the analysis data of inhibition or enhancement of H_2_ production by individual antibiotic.

**Table 2:** Raw and analytical data of H_2_ yield changes due to the application of treatment on various dosage of single antibiotics. Table 2-1 Raw data of H_2_ yield changes due to the application of treatment on various dosage of single antibiotics.

**Table:** **Table 2.***(Continued)*

**Table 2-2:** ST data of H_2_ yield changes due to the application of treatment on various dosage of single antibiotics

**Table 2-3:** ST-analysis of H_2_ yield changes due to the application of treatment on various dosage of single antibiotics.

**Table 3:** Raw and analytical data of H_2_ yield changes due to the application of treatment on various dosage of compound antibiotics **Table 3-1** Raw data of H_2_ yield changes due to the application of treatment on various dosage of compound antibiotics

**Table:** **Table 2.***(Continued)*

**Table 3-2:** ST data of H_2_ yield changes due to the application of treatment on various dosage of compound antibiotics

**Table 3-3:** ST-analysis of H2 yield changes due to the application of treatment on various dosage of compound antibiotics.

Recording the raw and statistical data of Aldehyde Group (CHO) modification with the application on differ dosage of single and compound antibiotics respectively in Tables 4 and 5. Each treatment contains of 5 parallel samples. C_CHO is the CHO concentration and the unit is mM/L; ST_CHO is the average CHO concentration for reach treatment and the unit is mM/L.

**Table 4:** Data of Aldehyde Group (CHO) modification with the application on differ dosage of single antibiotics.

**Table 5:** Data of Aldehyde Group (CHO) modification with the application on differ dosage of compound antibiotics.

Tables 6 and 7 documented the raw and statistical data of pH by utilising diverse treatment on distinct dosage of single and compound antibiotics respectively. Each treatment consist of 5 parallel samples. V_pH is the pH value of samples solution and ST_pH is the average pH for each treatment.

**Table 6:** Data of pH by utilising diverse treatment on distinct dosage of single antibiotics.

**Table 7:** Data of pH by utilising diverse treatment on distinct dosage of compound antibiotics.

Tables 8 and 9 record the raw and statistical data of OD_600_ with the approach on various dosage of single and compound antibiotics in correspondingly Respective treatment accommodated 5 parallel samples. V_OD_600_ specified the absorbance value of samples solution, it could be represented in the form of A. ST_ OD_600_ is the average absorbance value for each treatment and the unit is A.

**Table 8:** Data of OD_600_ with the approach on various dosage of single antibiotics.

**Table 9:** Data of OD_600_ with the approach on various dosage of compound antibiotics.

**Fig. 1:**
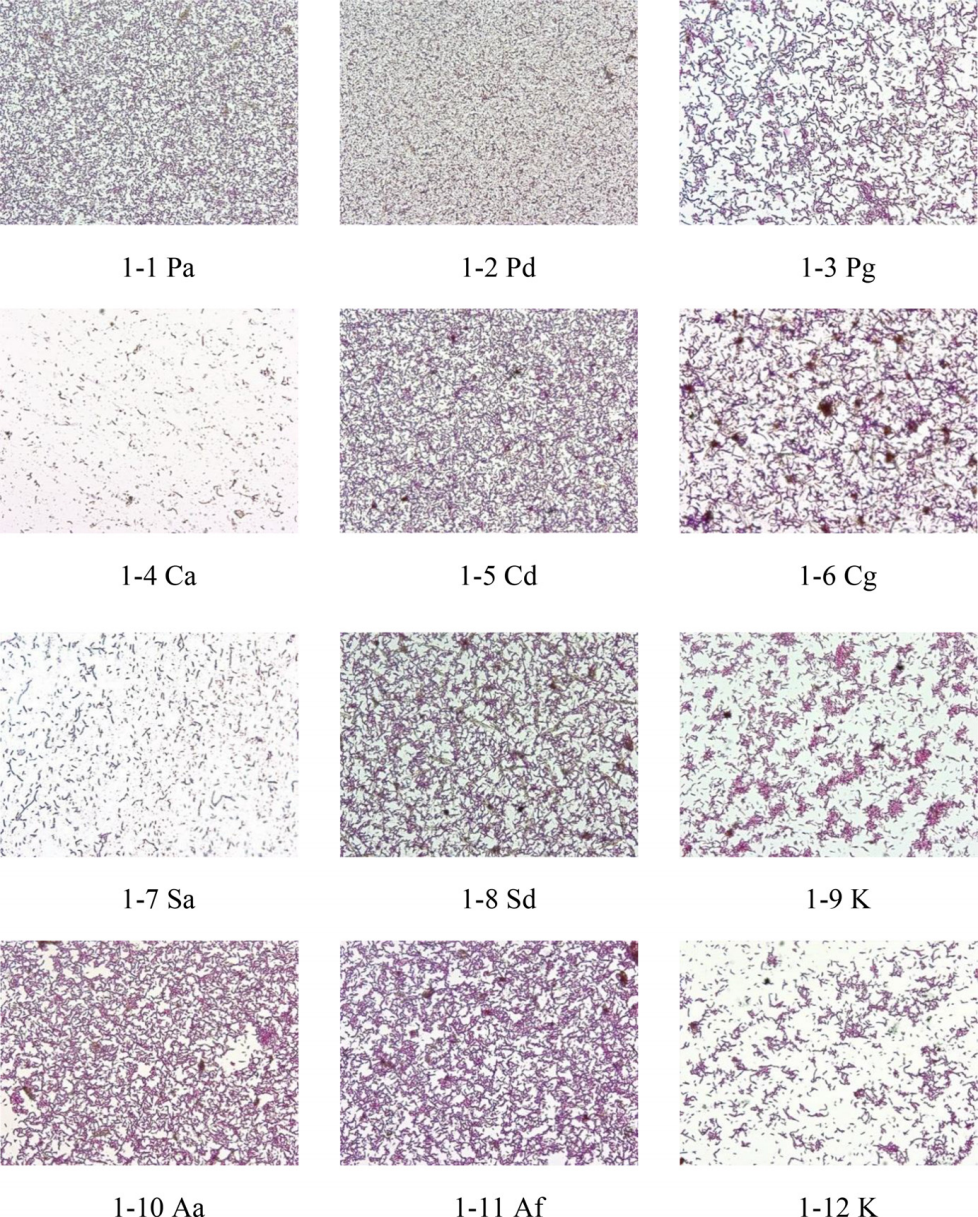

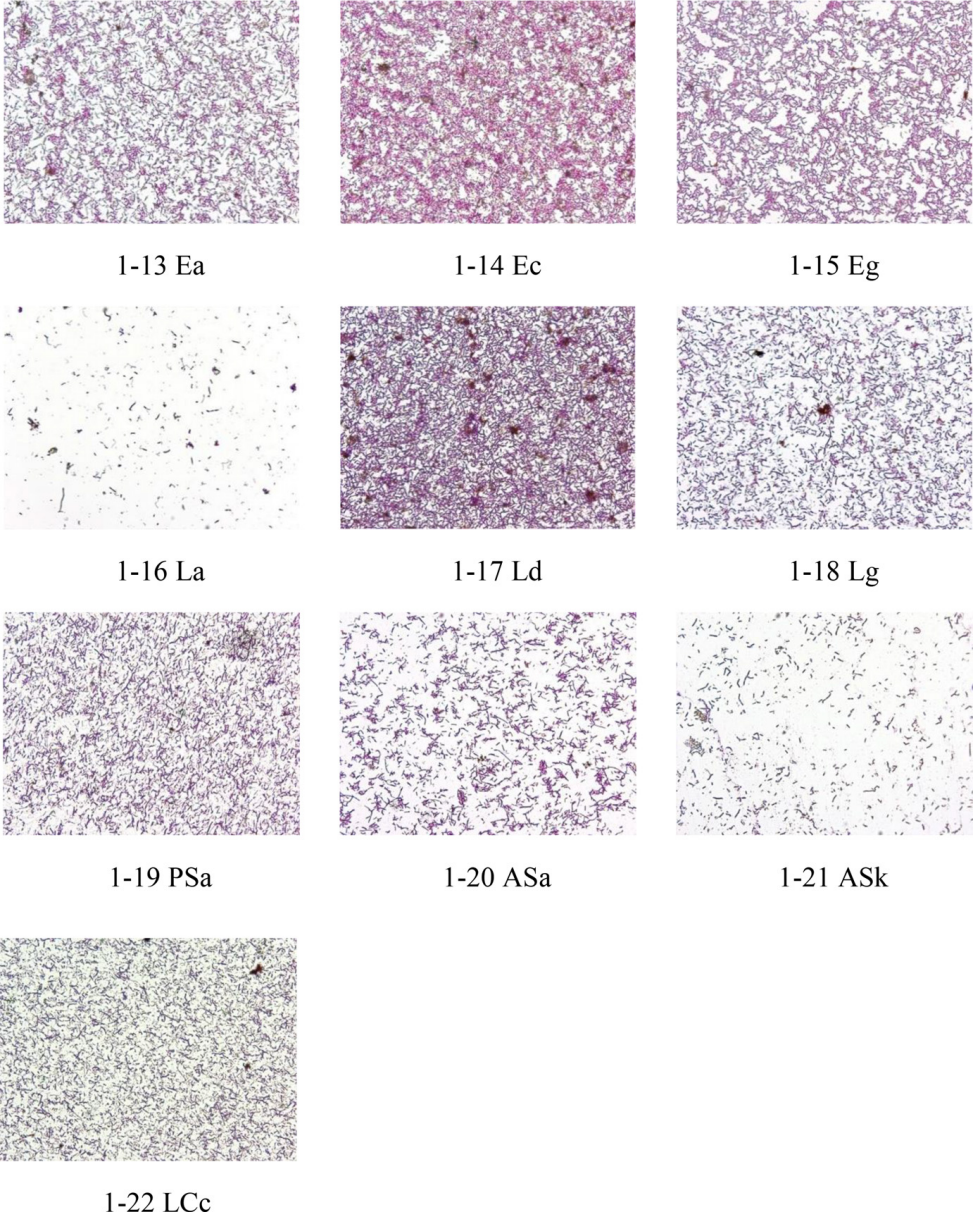
Gram staining of typical samples of single antibiotics and compound antibiotics with 40 × objective lens.

Images in Fig. 1 are gram staining of typical samples of single and compound antibiotics. In the below microscopy image gallery, Fig. 1-1 to 1-18 refers to the gram test photo of single antibiotic treatment samples; Fig. 1-19 to 1-21 refers to the gram test photo of compound antibiotic treatment samples. The objective lens magnification used for all images was 40 × .

**Fig. 2:**
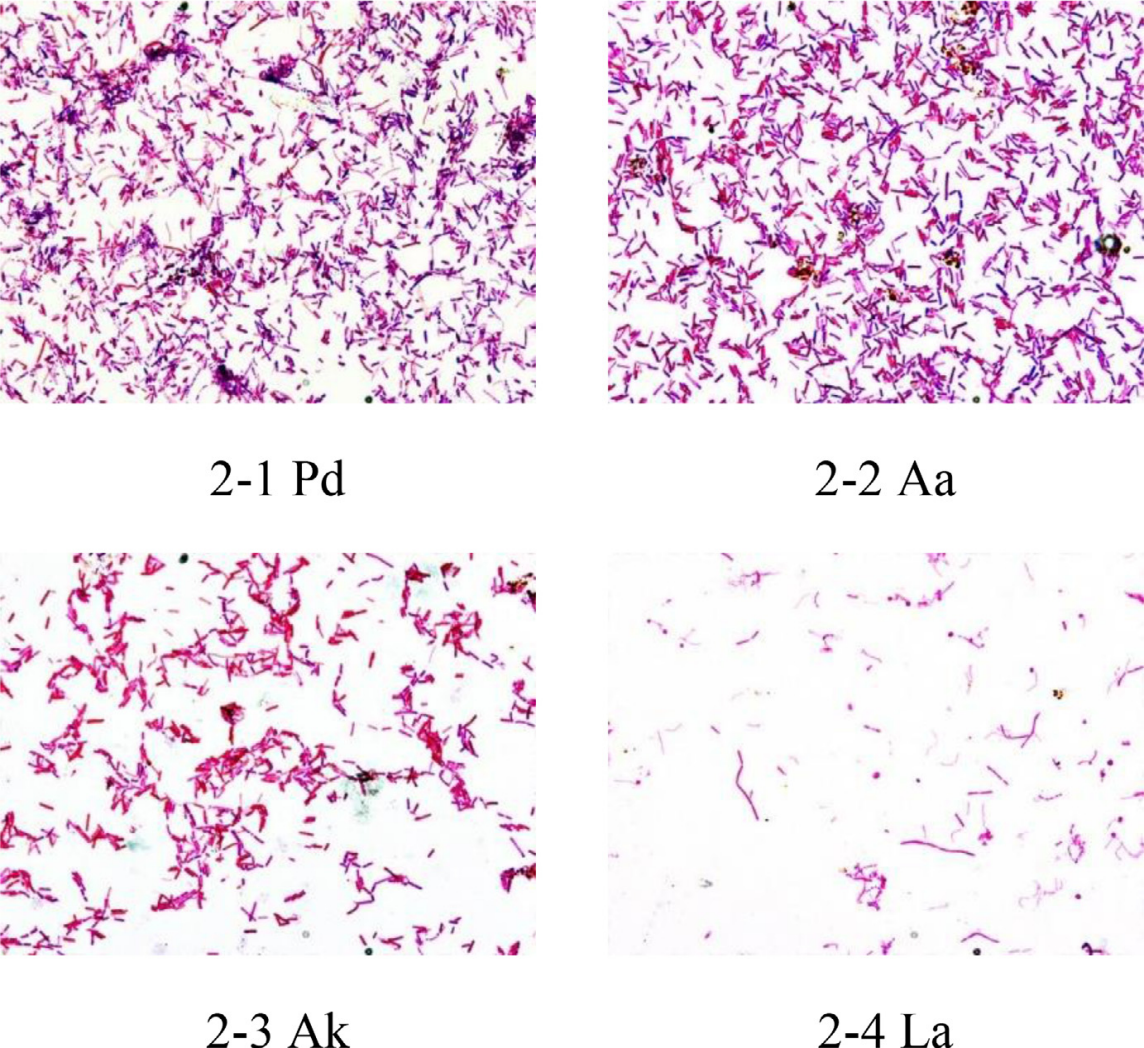
Gram staining of typical samples with 100 × objective lens.

Images in Fig. 2 are gram staining of typical samples of Penicillin G, Amikacin Sulfate, Levofloxacin treatment groups. The objective lens magnification used for all images was 100 × . The correspondence between photos and samples is shown in Table 10.

**Table 10:** Sample table of gram test photographs.

The correspondence between photos (Fig. 1 and Fig. 2) and sample numbers is shown in Table 10.

**Fig. 3:**
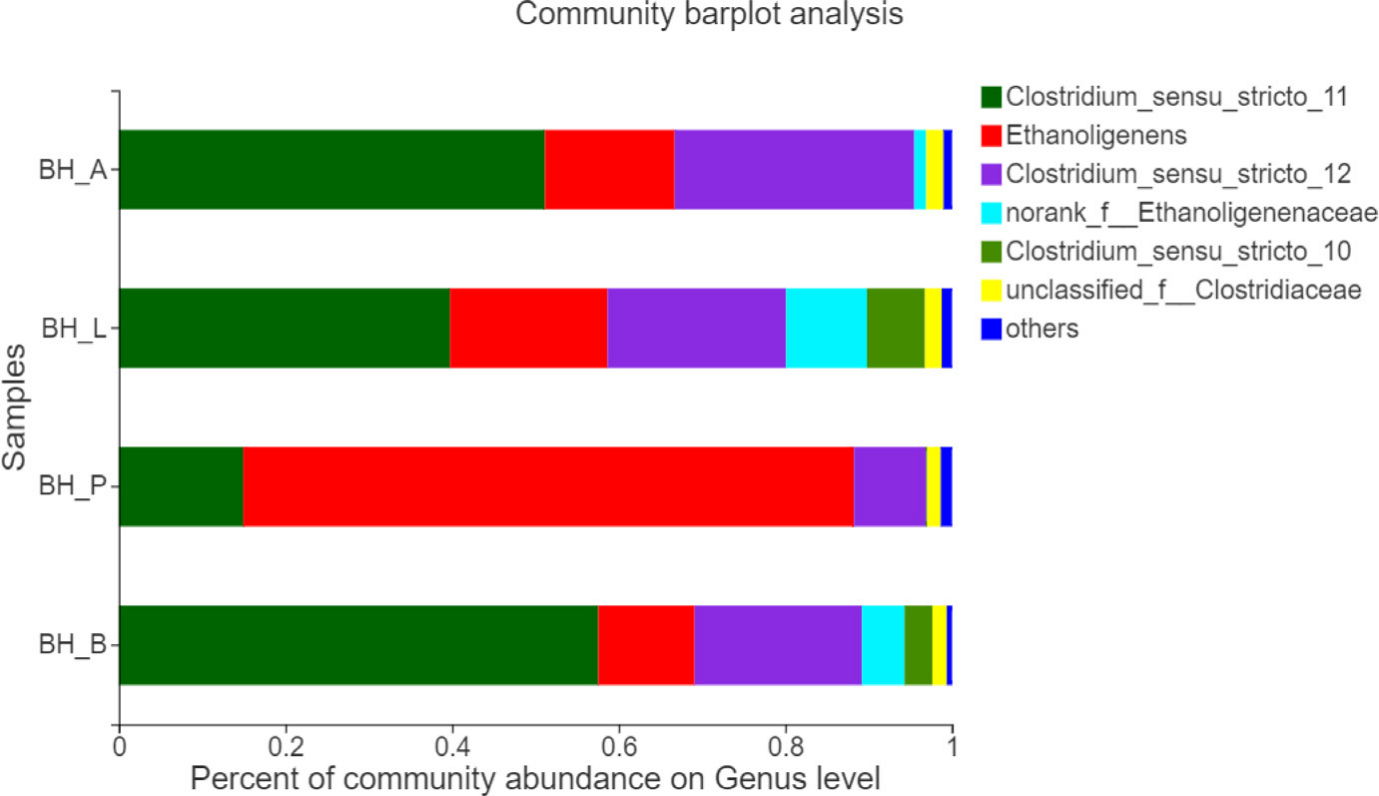
Community bar chart.

**Fig. 4:**
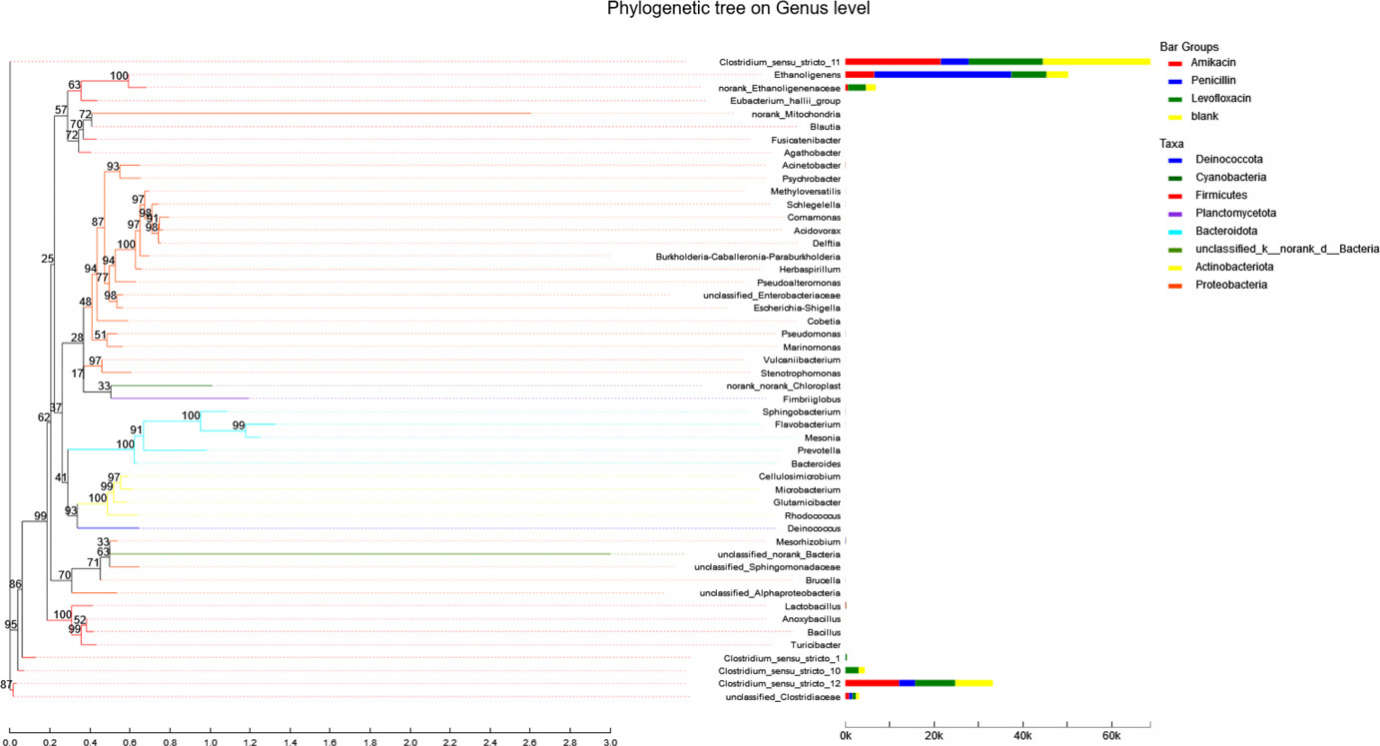
The evolutionary tree.

**Fig. 5:**
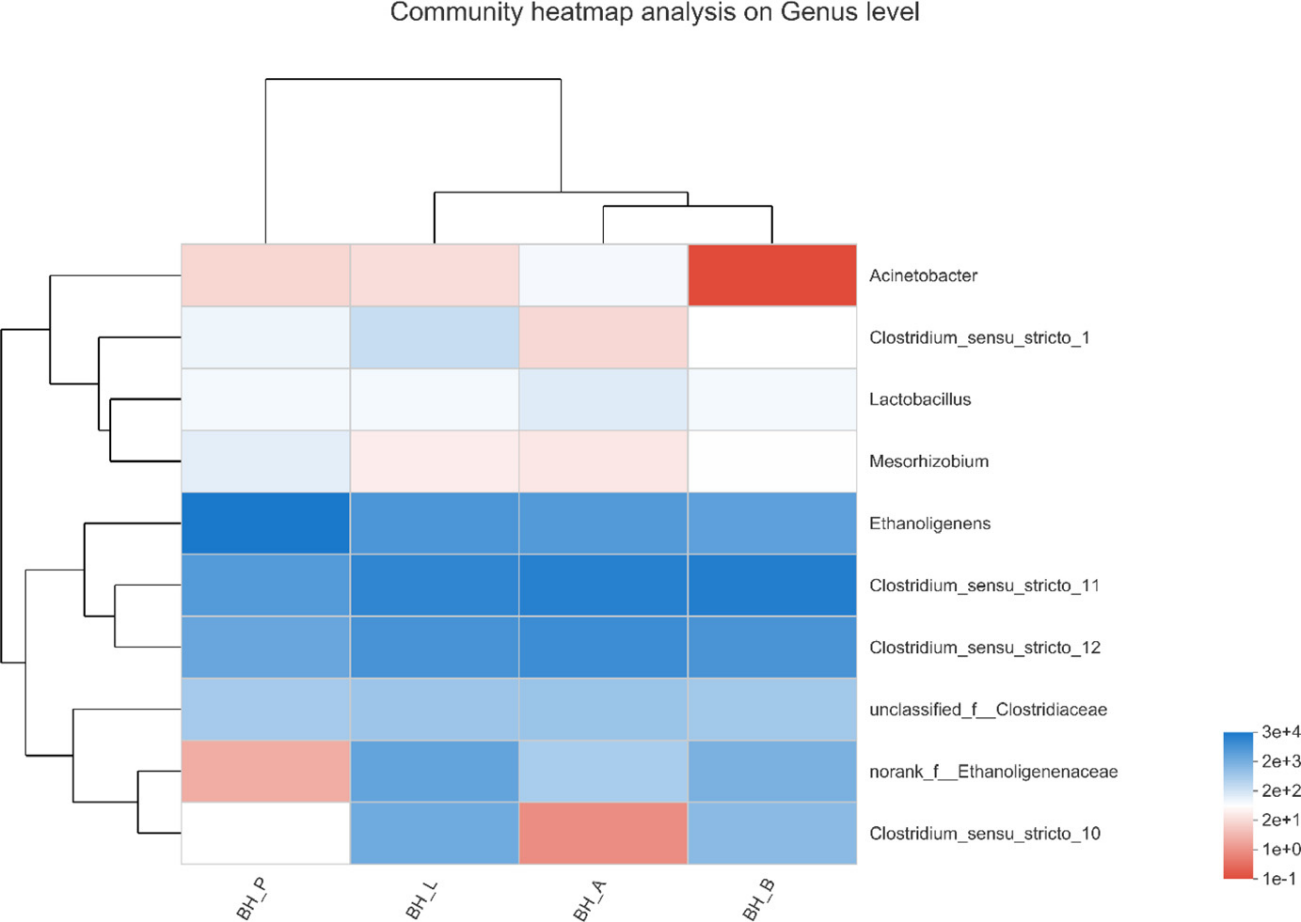
Top 10 species heatmap.

**Fig. 6:**
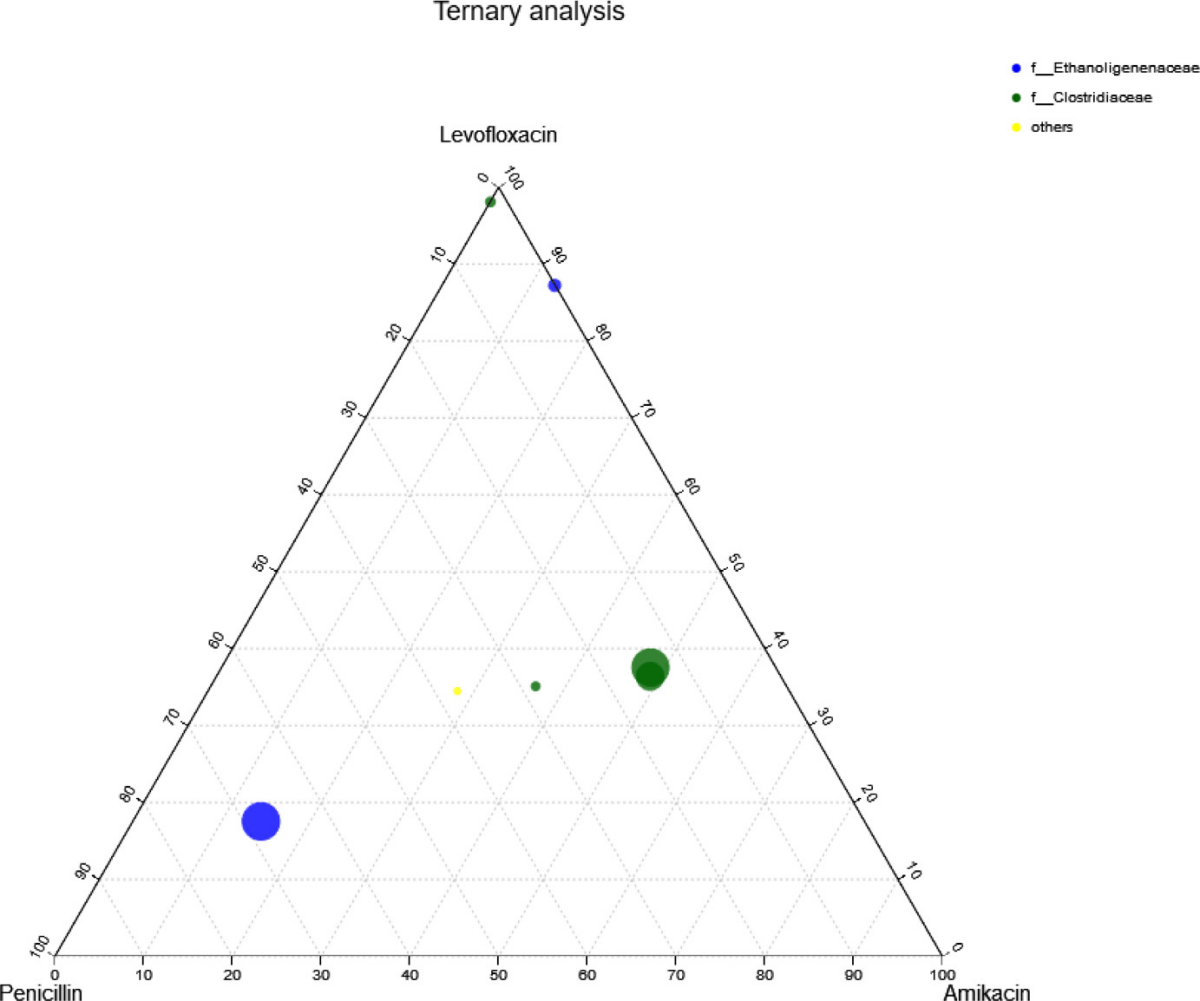
Ternay analysis.

**Fig. 7:**
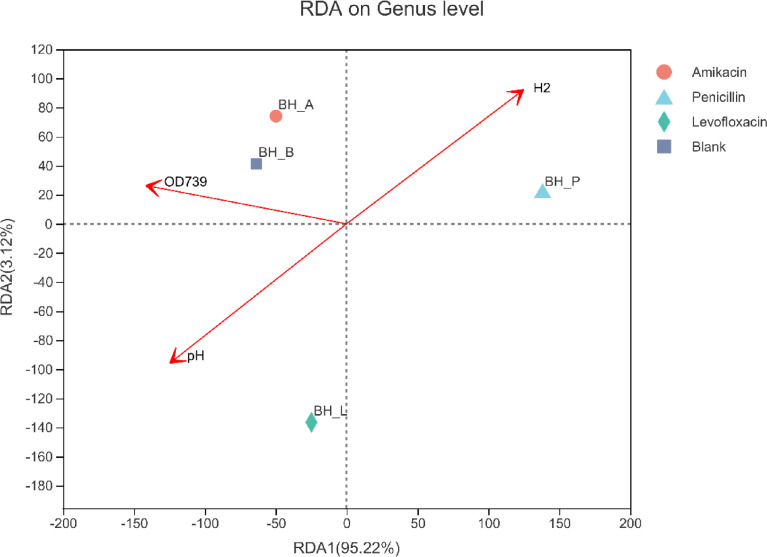
RDA-CCA analysis.

**Fig. 8:**
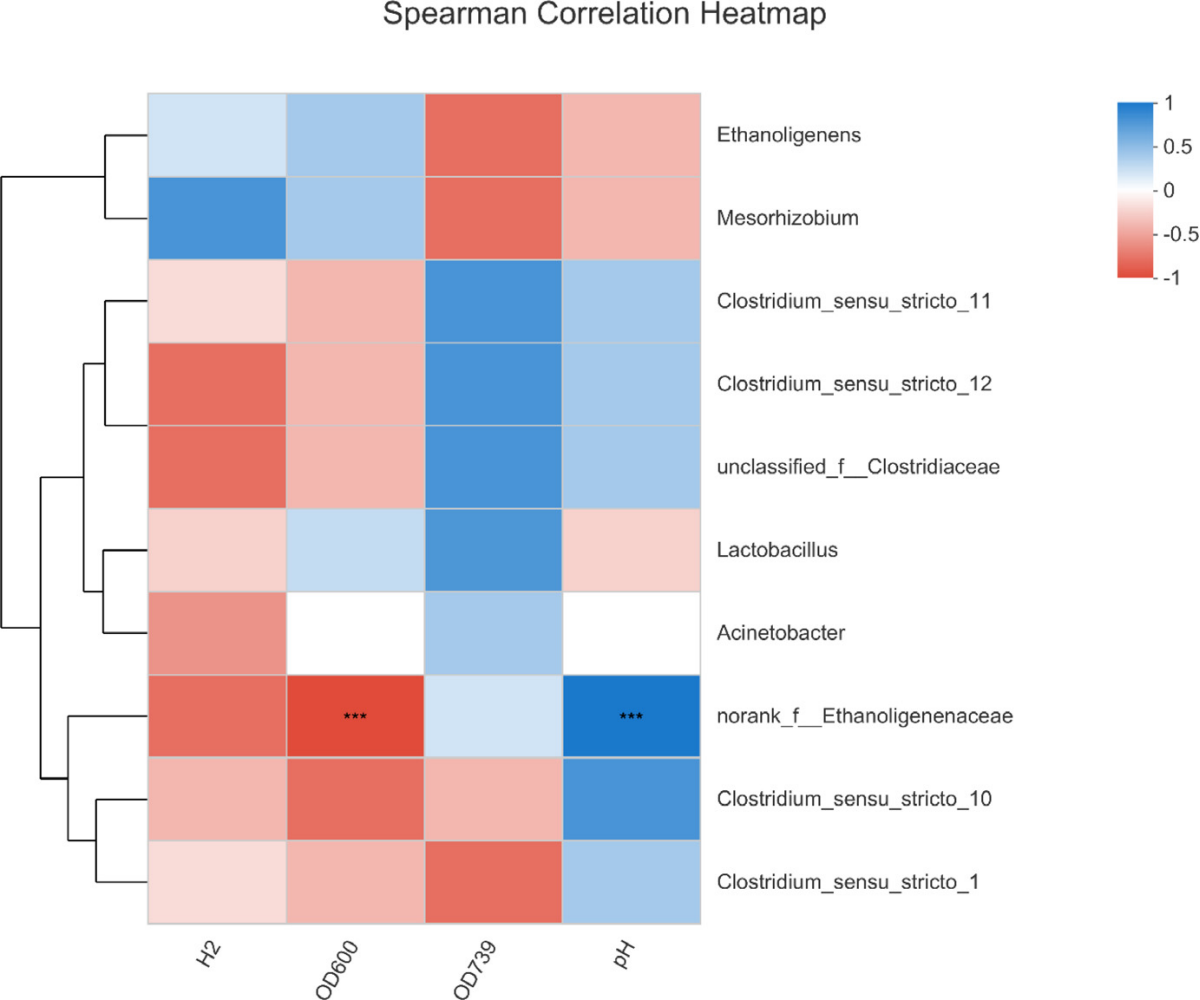
Spearman correlation heatmap.

**Fig. 9:**
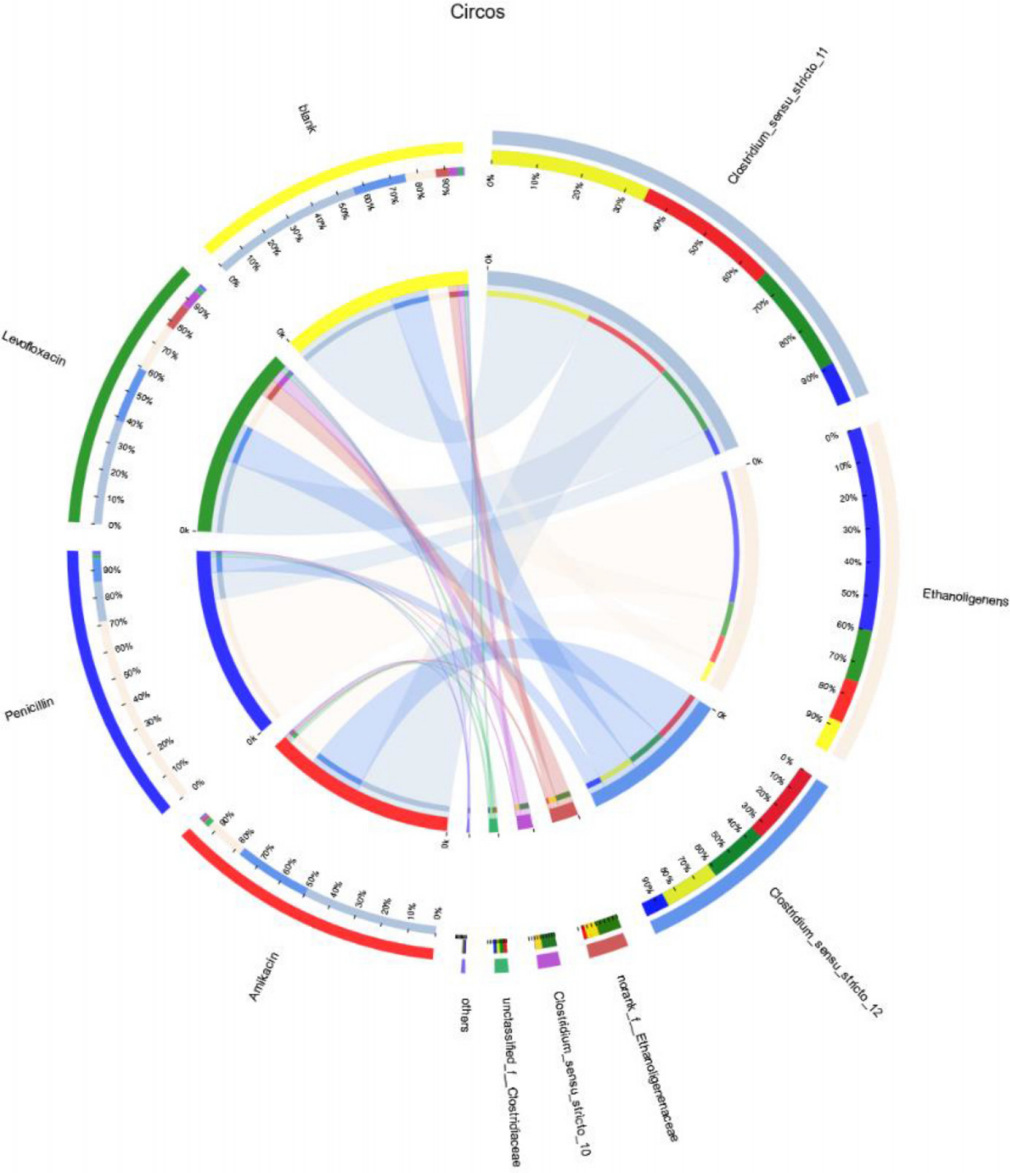
Circos analysis.

Based on the biodiversity test of Pd, Aa, La and K samples (The 16S rRNA gene sequences were deposited in the NCBI Sequence Read Archive under accession number PRJNA784035), a series of species composition analysis and correlation analysis were carried out and data mapping were performed. Analysis and mapping include: bar chart of the distribution of microbial diversity (Fig. 3), evolutionary tree on Genus level (Fig. 4), community heatmap analyais on Genus level (Fig. 5), Ternary analysis (Fig. 6), spearman correlation heatmap of bacterial on Genus level (Fig. 7), circos graph of the correspondence between samples and species (Fig. 8), PDA-CCA analysis of the correlation between Penicillin G, Amikacin Sulfate, Levofloxacin treatment and CHO, pH, H_2_ yields (Fig. 9).

## 2 Experimental Design, Materials and Methods

### 2.1 Medium and Culture Conditions

Coal geology H_2_-producing bacteria community was isolated from enrichment samples collected from an anthracite sample extracted in Zhaozhuang coal mining located in Jincheng, Shanxi Province (GPS coordinates is 35°34′10″N, 112°53′55″E). The H_2_-producing bacteria were grown anaerobically on Potato Dextrose medium (abbreviated as PD medium)[1]. The content of the PD medium was (g/L): potato soluble starch, 20.00; dextrose, 20.00; NH_4_Cl, 3.50; KCl, 3.20; NaCl, 0.70; MgSO_4_•7H_2_O, 0.20, FeCL_3_, 0.05; CaCl_2_, 0.02; yeast extract, 0.50, and 1.00 mL/L of C_12_H_7_NO_4_ was added as an oxygen indicator [1]. Final medium pH=6.2. The prepared PD medium was sterilized at 121°C and 0.105 MPa for 25 minutes. The PD medium was then mixed with the bacterial solution at a ratio of 4:1 in an anaerobic chamber (A 95, WDS, Britain). The mixed medium was divided into 200 mL aliquots to anaerobic culture flasks, then sealed with butyl rubber stoppers and removed from the chamber. The samples configured in accordance to experimental design and were placed in shakers (JK-LI-15, Jingke, China) with temperature set at 40 °C with a shaking speed set at 60 rpm [2]. Cultivation time: 3 days.

### 2.2 Selection of Antibiotics and Gradient Experiment Design

Six antibiotics used in experiments comprise Penicillin G, Cefaclor, Streptomycin Sulfate, Amikacin Sulfate, Erythromycin, and Levofloxacin. The maximum dosage (abbreviated as MD) for each antibiotic was referred to the highest concentration in urine which was recorded in the instructions.

The gradient percentage (abbreviated as GP) of single antibiotic was set by dichotomy method from 100% to 1%, which were: 100.00%, 50.00%, 25.00%, 12.50%, 6.25%, 3.13%, 1.56%. The compound antibiotic concentration grade was set as 100.00%, 25.00%, 12.50%, 6.25%. The corresponding relationship between each antibiotic gradient and dosage is provided in Table 2.

Meanwhile, 0.00% comparison group was set for each antibiotic. 5 parallel samples were set for each antibiotic and each concentration.

### 2.3 H_2_ Yield Data Collection

The gas yield of each sample was collected through 1500 mL gas sampling bags. The total gas yield (record as V_t_) was tested with 100 mL gas needle at the end of each experiment. The H_2_ yield was calculated on the base of the total gas yield and H_2_ concentration (formula 1).

Gas composed of H_2_ was analysed by using an Agilent 7890A gas chromatograph. The column was Agilent Carbonplot (60 m × 320 um) and the carrier gas is high purity nitrogen (99.999%). The carrier gas flow rate was set at 3 mL/min. The injection port was maintained at 150 °C, the oven temperature was 25 °C, the TCD was operated at 200 °C, reference flow rate 400 mL/min, tail flow rate 8 mL/min. The retention time for H_2_ was 3.2 minutes, and CO_2_ was 4.4 minutes [1]. Calibration standards consisting of 40% H_2_, 20% CO_2_, 10% CH_4,_ and 30% N_2_ were injected to generate the calibration plot. Each sample gas composition test was repeated 3 times. The average value of the three test results was recorded as the original data of the H_2_ concentration of the sample. The H_2_ concentration was recorded as C_H2_.

The H_2_ yield was calculated as follow (formula 1):

(1) $$M_{H_{2}}=\frac{V_{T}\times C_{H_{2}}\times \;273.15}{22.4\;\times \left(W_{s}\times 0.20\right)\times \left(273.15+T_{r}\right)}$$

Where: M_H2_: molar amount of H_2_ (mM);

V_T_: total gas yield for each sample (L);
C_H2_: H_2_ concentration for each sample (%);
T_r_: ambient temperature (°C);
W_s_: the content of potato soluble starch in medium (g/L).

The total gas production (T_Gas), H_2_ concentration (C_H_2_), H_2_ production (V_H_2_), average H_2_ production rate (ST_H_2_) and deviation, and substrate molar H_2_ production rate (Mm_H_2_) and deviation of each experimental group, show in Table 3 and Table 7.

Calculation method of average H_2_ production rate: After removing the maximum or minimum deviation from each group, the average H_2_ production was calculated with remaining 4 data.

Calculation method of deviation value: Calculated from the average H_2_ production value and all 5 data in each group.

### 2.4 CHO Molarity Data Collection

In the completion stage of each experiment, the samples of every group were re-randomized thus CHO was determined.

The CHO molarity in each sample was measured with Benedict's test method. 2 mL sample was mixed with 0.5 mL Benedict's reagent in a clean test tube. And the solution was heated in a boiling water bath for 5 minutes. Immediately after the solution was ultrasonically diffused, the absorbance was measured at 739 nm by spectrophotometer (defined as OD_739_) (BioMate 3S, Thermo Scientific, America). OD_739_ is correlated with CHO molarity. Glucose was used as calibration standards consisting of (mM) 5.00, 2.50, 0.50, 0.25, 0.05, 0.025, and 0.010 were measured to generate the calibration plot. Each gas composition test sample was repeated 3 times. The average value of the three test results was recorded as the original data of the CHO concentration of the sample.

The CHO concentration (C_CHO) and average CHO concentration (C_CHO) and deviation show in table 4 and table 8.

Calculation method of average CHO concentration: the average CHO concentration was calculated with the 4 samples in each group which selected in the calculation of average H_2_ production.

Calculation method of deviation value: Calculated from the average CHO concentration value and all 5 data in each group.

### 2.5 pH Data Collection

The samples of every groups were re-randomized and then pH was measured.

15 mL of culture medium was centrifuged at 12000 × g for 5 minutes (SL 16A, Thermo Scientific, America), and the supernatant used to test pH value. The pH level of each sample has been measured by pH meter (Star A211, Orion, America). Each test sample was repeated 3 times. The average value of the three test results was recorded as the original data of the pH of the sample.

The pH value (pH) and average pH (ST_Ph) and deviation show in Table 5 and Table 9.

Calculation method of average pH value: the average pH value was calculated with the 4 samples in each group which is being selected in the calculation of average H_2_ production.

Calculation method of deviation value: Calculated from the average pH value and all 5 data in each group.

### 2.6 OD_600_ Data Collection

The samples of every groups were re-randomized and then OD_600_ was measured.

OD_600_ was measured at 600 nm by spectrophotometer (BioMate 3S, Thermo Scientific, America). OD_600_ test for each sample was repeated 3 times. The average value of the three test results was recorded as the original data of the OD_600_ value of the sample. A blank culture medium containing no starch was used as a blank sample to zero the spectrophotometer.

The OD_600_ value (OD600) and average pH (ST_OD600) and deviation show in table 6 and table 10.

Calculation method of average OD_600_ value: the average OD_600_ value was calculated with the 4 samples in each group which selected in the calculation of average H_2_ production.

Calculation method of deviation value: Calculated from the average OD_600_ value and all 5 data in each group.

### 2.7 Gram Stain Test and Bacterial Morphology Observation

The method of gram stain was used to distinguish and classify bacterial species, gram-positive bacteria, and gram-negative bacteria, based on the physical properties of cell walls. The microbial density of the gram stain was observed at 40 × and 100 × objectives (BX43, Olympus, Japan) and photos taken.

According to the variation of H_2_ production under different kinds of signal antibiotic treatment, 3 representative sample were selected in each group, and each sample retained 1 representative photograph with 40 × objective lens. For compound antibiotic treatment groups, 1 representative sample were selected in each group, and each sample retained 1 representative photograph with 40 × objective lens (Table 10, Fig 1-1 to 1-20).

In addition, 1 photograph was taken for each the biodiversity analysis samples with 100 × objective lens.

### 2.8 DNA Extraction and PCR Amplification

(1) 10 mL of cultured medium in each sample was collected at the end of the experiment. Bacteria was concentrated to 1 mL by centrifugation (SL 16A, Thermo Scientific, America) and stored in cryovials at -80 °C (DW-86L728J, Haier, China). The centrifugal force was set to 13000 × g, and centrifuged for 10 minutes.
(2) Total genomic DNA was extracted from 1 mL concentrated underground water samples using E.A.N.A. Soil DNA Kit (OMEGA, Georgia, GA, America) according to manufacturer's instructions. The final DNA concentration and purification were determined by spectrophotometer (NanoDrop 2000 UV-vis, Thermo Scientific, America), and DNA quality was checked by 1% agarose gel electrophoresis.
(3) The V3-V4 hypervariable regions of bacteria 16S rRNA gene was amplified with primers 338F (5′- ACT CCT ACG GGA GGC AGC AG - 3′) and 806R (5′- GGA CTA CHV GGG TWT CTA AT - 3′) by thermocycler polymerase chain reaction (PCR) (GeneAmp 9700, ABI, America) [3]. The DNA amplification was performed using the following program: 3 min of denaturation at 95°C, 27cycles of 30 s at 95°C, 30 s for annealing at 55°C, and 45 s for elongation at 72°C, and a final extension at 72°C for 10 min [4]. PCR reactions were performed in triplicate 20 μL mixture containing 4 μL of FastPfu Buffer, 2 μL of 2.5 mM dNTPs, 0.8 μL of each primer (5 μM), 0.4 μL of FastPfu Polymerase and 10 ng of template DNA.
(4) The result PCR products were extracted from a 2% agarose gel and further purified using the AxyPrep DNA Gel Extraction Kit (Axygen Biosciences, Union City, CA, America) and quantified using QuantiFluor^TM^-ST (Promega, America).
(5) Illumina MiSeq sequencing

 Purified amplicons were pooled in equimolar and paired-end sequenced (2 × 300) on an Illumina MiSeq sequencing (Illumina, San Diego, America) according to the standard protocols by Majorbio Bio-Pharm Technology Co. Ltd (Shanghai, China). The 16S rRNA gene sequences were deposited in the NCBI Sequence Read Archive under accession number PRJNA784035.

(6) Process of sequencing data

 Raw fastq files were demultiplexed, quality-filtered by Trimmomatic and Merged by FLASH with the following criteria:
  1) The reads were truncated at any site receiving an average quality score <20 over a 50 bp sliding window;
  2) Primers were exactly matched allowing 2 nucleotide mismatching, and reads containing ambiguous bases were removed;
  3) Sequences with overlap longer than10 bp were merged according to their overlap sequence.
(7) Operational taxonomic units (OTUs) were clustered with 97% similarity cutoff using UPARSE (version7.1 http://drive5.com/uparse/) and chimeric sequences were identified and removed using UCHIME. The taxonomy of each 16S rRNA gene sequence was analyzed by RDP Classifier algorithm (http://rdp.cme.msu.edu/) against the Silva (SSU123) 16S rRNA database using confidence threshold of 70%.

### 2.9 Microbial Diversity and Correlation Analysis with Environmental Factors

Community column chart, with respect to the results of taxonomic analysis, the species composition at the genus level of the four samples was calculated. Software: Based on the data table in the tax_summary_a folder, use the R language (version 3.3.1) tool (Fig. 3).

The evolutionary tree selects the top 50 species in the total abundance of the species taxonomic level, uses ML (Maximum likelihood) for construction, presents the phylogenetic relationship of the species in the form of a ring diagram. Software: Mega (version 10.0 https://www.megasoftware.net/) (Fig. 4).

Heatmap mapping adopted the top 10 species of Species level, the second classification level: Phylum, and the species hierarchical clustering method: average. Software and algorithms: R language (version 3.3.1) vegan package (Fig. 5).

Ternary phase diagram for comparative analysis of the species composition of the three samples based on taxonomic information. Taxonomy level: genus; Combined calculation method of samples within a group: average value; Color level: family. Software: GGTERN (http://www.ggtern.com/) (Fig. 6).

RDA analysis is a PCA analysis constrained by pH, CHO (OD_739_) and H2 yield rate factors, which combines corresponding analysis with multiple regression analysis, each step of the calculation is regressed with environmental factors. RDA based on a linear model and CCA based on a unimodal model (Fig. 7).

1) Selection principle of RDA or CCA model: initially employing species-sample data (sample OTU table with 97% similarity) to undertake DCA analysis, examine the size of the first axis of Lengths of gradient in the analysis result, hypothetically assuming that it is greater than or equal to 3.5, it could be assumed as CCA, granted that it is less than 3.5, the result of RDA is better than that of CCA.
2) Determine the maximum Pearson correlation coefficient of the distribution difference between environmental factors and sample communities through the bioenv function, obtain a subset of environmental factors through the maximum correlation coefficient.
3) Perform CCA or RDA analysis on the sample species distribution table and environmental factors or environmental factor subsets respectively.
4) Judging the significance of CCA or RDA analysis by permutest analysis similar to ANOVA.

Software: R language (version 3.3.1) RDA or CCA analysis and graphing in the vegan package.

Spearman correlation heatmap, calculate the Spearman rank correlation coefficient between H_2_ yield rate, OD_600_, CHO (OD_739_), pH with the top 10 species of Genus level, and the obtained numerical matrix dispalys by Heatmap. Software: R (version 3.3.1) (pheatmap package) (Fig. 8).

The Circos chart was drawn using the Genus taxonomy level, and the abundance of the samples in the group is calculated by summing up, and the relative abundance >0.01. Software: Circos-0.67-7 (http://circos.ca/) (Fig. 9).

## Ethics Statements

This work involves the research based on the response law of H_2_ yield capacity of H_2_-producing bacteria to different types and dosages of antibiotics. This manuscript presents datasets that are the authors’ original work and co-submitted with the manuscript “The response regularity of bio-hydrogen production by anthracite H_2_-producing bacteria consortium to six conventional veterinary antibiotics” (https://doi.org/10.1016/j.jenvman.2022.115088) and is not currently being considered for publication elsewhere. The paper reflects the authors’ own research and analysis in a truthful and complete manner. In addition, the paper properly credits the meaningful contributions of co-authors and co-researchers. All sources used are adequately disclosed. All authors have been personally and actively involved in substantial work leading to the paper and will take public responsibility for its content.

## CRediT Author Statement

**Dong Xiao:** Conceptualization, Writing – Original draft preparation, Funding acquisition; **Hailun He:** Writing – review & editing, Funding acquisition; **Xiaoxin Yan:** Conceptualization, Writing – review & editing; **Norberto Daniel Diaz:** Methodology, Data Curation, Funding acquisition; **Dayong Chen:** Data Curation; **Jing Ma:** Data Curation; **Yidong Zhang:** Visualization, Data Curation; **Jin Li:** Visualization; **Mohamed Keita:** Data Curation, Writing – review & editing; **Essono Oyono Julien:** Data Curation, Writing – review & editing; **Xiaotao Yan:** Data Curation.

## Declaration of Competing Interest

The authors declare that they have no known competing financial interests or personal relationships that could have appeared to influence the work reported in this paper.

## Data Availability

Antibiotic sensitivity test of hydrogen-producing bacteria (Original data) (National Library of Medicine). Dataset of response characteristics of H_2_-producing bacteria consortium to β-Lactams, Aminoglycosides, Macrolides, Quinolones antibiotics (Original data) (Mendeley Data).
